# Child Vaccination Status and Behavioral and Social Drivers of Vaccination Among Their Caregivers in the Philippines: Cross-Sectional Survey Study Comparison of Household, Mobile, and Online Modes

**DOI:** 10.2196/81059

**Published:** 2026-04-10

**Authors:** Kimberly E Bonner, Mikka Hipol, Dominique Sy, Rivandra Royono, Douglas Johnson, Isabel del Rosario, Alice Redfern, Darahlyn Biel-Romualdo, Man Kai Wong, Eugene Lam, Shibani Kulkarni, Kirsten Ward, Romel Lacson, Meng-Yu Chen, James Matthew Miraflor, Devon Ray Pacial, Rowena Bunoan, Hugo Catan, Talya Shragai

**Affiliations:** 1 Global Immunization Division, Centers for Disease Control and Prevention, 1600 Clifton Rd NE, Atlanta, GA, 30329, United States, 1 4706931312; 2 IDInsight, Makati, Philippines; 3 Centers for Disease Control and Prevention, Global Health Center, Atlanta, GA, United States; 4 The Task Force for Global Health, Atlanta, GA, United States; 5 Republic of the Philippines Department of Health, Manila, Philippines

**Keywords:** mHealth, low- and middle-income countries, health surveys, reproducibility of results, data collection methods, vaccination, behavioral and social drivers, mobile surveys, mobile health

## Abstract

**Background:**

The World Health Organization recommends that countries routinely collect data on the behavioral and social drivers (BeSD) of vaccination to inform public health interventions that increase vaccine uptake. There is a need to identify data collection methods that can rapidly and inexpensively collect representative data, particularly in low- and middle-income countries.

**Objective:**

This study aimed to understand BeSD drivers of vaccination in the Philippines and assess the trade-offs between survey methods. We compared responses to household, mobile, and online surveys in terms of demographics, vaccination status, responses to BeSD questions, and cost.

**Methods:**

We conducted concurrent household, mobile (SMS text messaging and interactive voice response), and online surveys among caregivers of children 2 years of age and below in Regions V and XII of the Philippines, with sampling differing by survey method. We assessed, for each survey method, (1) respondent demographics (sex, age, region, and socioeconomic status) and (2) the weighted proportion of responses from caregivers of children who received at least one dose of diphtheria-pertussis-tetanus (DPT)–containing vaccine. We estimated the weighted proportion of each BeSD survey response option and calculated the financial cost (monetary outlays) per survey response from an implementer’s perspective by summing the costs incurred in each survey method and dividing by the number of responses received.

**Results:**

We surveyed a total of 1201 household respondents, 2153 mobile respondents, and 398 online respondents from January to March 2025. We found that online and mobile survey respondents were more likely to be male and have completed high school than household survey respondents. The weighted proportion of respondents indicating that their child had received at least one dose of DPT vaccine was 91.8% (n=1090; 95% CI 90%‐93.3%) for the household survey, 90.3% (n=1853) for the mobile survey, and 85% (n=346) for the online survey. With regard to vaccine demand, more than 85% of respondents in each survey method indicated that vaccines are very important, very safe, supported by family, and that they knew where to bring a child for vaccination. More than 30% of mobile and online survey respondents indicated that it was not easy to pay for vaccination. The financial cost to conduct the survey per survey response was US $2.61 for the online survey, US $6.93 for the mobile survey, and US $29.38 for the household survey.

**Conclusions:**

In the Philippines, household, mobile, and online survey methods reached caregivers of children who were unvaccinated against DPT, and these proportions were similar across survey methods. BeSD responses indicated high vaccine demand and challenges in caregivers’ cost to access vaccination. Determining the most appropriate survey method depends on trade-offs between representativeness and costs. However, areas with strong connectivity and high mobile device ownership can consider mobile and online methods as a lower-cost alternative to rapidly collect BeSD data.

## Introduction

In low- and middle-income countries (LMICs), there is a pressing need for methods of collecting health data beyond household surveys that can inform public health practice [1], particularly for measuring drivers of vaccine demand [2,3].

While household surveys are considered a gold standard for population-representative data [4], there are drawbacks to this method, including declining participation in urban areas, household survey costs, exclusion of nonstationary households [5], and infrequent periodicity [6]. Mobile phone surveys and online surveys offer promising alternatives to household surveys, particularly in areas with high mobile phone ownership rates and network penetration [7]. One key concern of both mobile and online surveys is that the population responding to these surveys might not be representative of the overall population of interest [8,9], and the responses to survey questions might differ by survey modality [10]. While some comparisons between remote data collection via self-administered SMS text messaging surveys have been made in LMICs, these assessments focused on nutrition indicators [10]. While mobile methods for social mobilization and vaccination reminders have been extensively employed in vaccination activities [11], few studies have examined bidirectional messaging or data collection.

The World Health Organization recommends that countries routinely monitor the behavioral and social drivers (BeSD) of vaccination at the community level using a series of validated questions derived from the BeSD framework [12]. An understanding of the drivers of vaccination in a population enables public health programs to develop tailored interventions that address specific population needs and concerns regarding vaccination. Of particular importance is understanding the needs of unvaccinated, also known as zero-dose, populations, defined as receiving zero doses of diphtheria-pertussis-tetanus (DPT)–containing vaccine. There is a need to identify other survey methods that can rapidly collect BeSD data at scale [13]. In the context of BeSD surveys, there is a need to understand how the proportion of respondents with unvaccinated and under-vaccinated children differs by survey method, including its associated costs.

The Philippines has high mobile phone penetration, widespread internet coverage, a high population of children who have not received recommended vaccines (zero-dose) [14], and documented barriers to vaccine demand [15]. Mobile phone ownership was 122 per 100 people [16], with an estimated 92% of households possessing at least 1 mobile phone in 2022 [17]. Cellular network coverage was 99%, and third generation of cellular network technology (3G) network access, which enables internet connectivity, was 96% in 2024 [18]. In addition, the Philippines has a longstanding community health worker system of Barangay Health Workers (BHWs), based in the smallest administrative unit (barangay). BHWs support primary care service delivery by maintaining contact with a designated list of families in their catchment area [19]. Self-administered mobile surveys distributed by BHWs offer an opportunity to leverage the potential speed and scalability of mobile data methods while garnering an improved response rate over random digital dial through the use of trusted BHW distribution networks. In addition, the Department of Health has established an online survey platform that can be readily accessed through a web search, Facebook, or Viber.

The overall objective of this study is to compare respondent characteristics and BeSD survey responses using 3 different data collection methods: household surveys, self-administered mobile surveys distributed via SMS text messaging by BHWs, and online surveys. Specific aims of this study are to (1) describe respondent demographics and vaccination status by data collection method, (2) compare how the distribution of responses to BeSD questions varies by survey method, and (3) describe the process of implementing the 3 survey methods, including financial costs from an implementer’s perspective and a data quality assessment of response accuracy in the mobile survey.

## Methods

### Recruitment

Participants for each survey method were recruited from 2 regions in the Philippines: Region V (Bicol) and Region XII (SOCCSKSARGEN). These regions were selected due to mobile penetration above 90% [17], a high population of children who received zero doses of DPT or with an incomplete vaccination series (30.1% unvaccinated in Region XII [17]; 64.7% under-vaccinated in Region V [20]), and security conditions that permit in-person data collection. Participants for each survey method were eligible if they were 18 years of age or older, were caregivers of a child 2 years old or younger, and were residents of Region V or XII during the survey period.

Sampling differed by survey method (Table 1). In the household survey, a 2-stage cluster sampling approach was used, drawing from the barangay, the smallest administrative units in each region. In first-stage cluster sampling, 91 barangays in Region V and 63 barangays in Region XII were selected using probability proportional to size, drawn from 2020 census data. The Philippines health system engages BHWs to maintain a comprehensive list of households in their catchment area to ensure that these households receive relevant health information and services. The second stage drew a simple random sample of 8 households with a child under 2 years of age from the BHW listing from each barangay, for a total target of 1200 households. The estimated sample size of 1200 for the household survey was calculated to ensure a margin of error on the outcome of interest of 5 percentage points or fewer, assuming 150 clusters, 8 households per cluster, an intraclass correlation of 0.3 on DPT vaccination status, and a control group mean of 0.5 (SD 0.5), based on the Demographic Health Survey estimate of 58% of children in these regions reported as fully immunized. Trained data collectors from the study team approached each of the randomly selected households in person and conducted an informed consent process with those who were eligible. If no eligible person was available at the household, data collectors visited each household a second time, and, if no eligible person was available again, they then randomly selected another household in the barangay as a replacement. Following informed consent, data collectors verbally administered a face-to-face 65-question survey in English, Tagalog, or an applicable regional language (including Cebuano/Bisaya, Hiligaynon, or Bikol), according to the preference of the participant. When available and with caregiver consent, data collectors verified the reported vaccination history by directly examining the child’s vaccine card. Deidentified data were recorded on a password-protected device, and survey responses were uploaded to a secure web server in accordance with the 2012 Data Privacy Act [21]. Participants with unvaccinated or incompletely vaccinated children were counseled to seek vaccination services from the nearest vaccination site.

**Table 1.** Summary of sampling, survey administration, survey questions, and weights by survey method: Philippines Regions V and VII, January-March 2025.

Survey method	Sampling	Administration	Number of questions	Analysis
Household	Two-stage cluster sampling: Stage 1: barangays selected using probability proportional to size. Stage 2: simple random sample of 8 households with a child under 2 y of age from the Barangay Health Worker (BHW) household listing.	In-person, interviewer-administered on a password-protected device.	65	Base weights for the probability of selecting each barangay and household based on probability proportional to size. Poststratification weights were used to match the marginal totals for strata defined by the combination of educational attainment of the household head, main water source, and region.
Mobile	Two-stage cluster sampling: Stage 1: barangays selected using probability proportional to size, drawn from 2020 census data. Stage 2: simple random sample of 2 BHWs per barangay who distributed survey invitation links to households listed in their catchment areas.	SMS text messaging invitation link sent by BHWs to eligible households in their catchment areas. Self-administered via SMS text messaging or interactive voice response, as selected by the participant.	15	Poststratification weights were used to match the marginal totals for strata defined by the combination of educational attainment of the household head, main water source, and region.
Online	Nonprobabilistic respondent-driven internet recruitment sampling; opt-in via Knowledge Informs Responsible Action chatbot.	Self-administered online survey	15	Poststratification weights were used to match the marginal totals for strata defined by the combination of educational attainment of the household head, main water source, and region.

In the mobile phone survey, barangays, the smallest administrative units in each region, were selected using probability proportional to size sampling in the same municipalities, but not the same barangays, as the in-person survey sites. A simple random sample of 2 BHWs per barangay was selected to distribute the mobile survey via SMS text messaging invitation link, using the BHW lists, which contained contact information, including mobile phone numbers, for each of the households with children 2 years of age and below. These BHWs were briefed on the purpose of the survey and asked to share an opt-in survey keyword via SMS text messaging with all eligible individuals using their catchment area household listing. To facilitate this outreach, each BHW received a one-time communication allowance of Php 300 (~US $5.40). As a quality check, BHWs were asked to report the total number of eligible individuals in their catchment area and the number of eligible individuals who had been sent the survey invitation message. The survey was hosted on the EngageSPARK platform, a self-service platform that sends interactive automated phone calls (interactive voice response [IVR] surveys) and 2-way SMS text messaging campaigns and collates survey responses. Recipients of the SMS text messaging survey invitation could opt into the survey by entering the survey shortcode included in the survey invitation to access the EngageSPARK platform. Following an initial screening questionnaire to confirm age, location, and whether there were children 2 years of age or younger in the household, participants were asked to proceed within the survey if they consented to participate. Next, participants could select to proceed in English or Tagalog and via SMS text messaging or by IVR.

In the online survey, we employed nonprobabilistic respondent-driven internet recruitment sampling, where regionally geo-targeted Facebook advertisements encouraged eligible respondents to opt into the survey. Respondents could opt into participating in the online survey through the pre-existing Knowledge Informs Responsible Action chatbot, known locally as *Katuwang na Impormasyon para sa Responsableng Aksyon*, hosted by the Philippines Department of Health (DOH) [22] and maintained by AI4GOV. The Knowledge Informs Responsible Action chatbot service offers information, collects survey data, and can be accessed through the DOH webpage, DOH Facebook page, and via Viber.

### Measures

Across the 3 surveys, we used consistent measures to ascertain eligibility, respondent demographics, child vaccination status, and responses to the BeSD questions.

The interviewer-administered household survey included 16 questions on caregiver demographics, 28 on child demographics and vaccination status, and 21 BeSD questions (Table S1 in Multimedia Appendix 1). Each survey method used consistent questions, and the household survey included additional questions at the behest of local authorities.

The self-administered mobile survey consisted of 15 questions, including 4 demographic questions, 7 core BeSD of vaccination questions, and 4 questions on demographics and vaccination status of the youngest child under 2 years of age in the household (Table S1 in Multimedia Appendix 1). We opted to assess a subset of the household survey questions, in alignment with the number and estimated completion time of other mobile surveys [7].

The self-administered online survey began with a welcome, eligibility confirmation, and informed consent process. Participants completed the 15-question survey in English, Tagalog, or Cebuano/Bisaya (Table S1 in Multimedia Appendix 1). The survey consisted of 4 demographic questions, 7 core BeSD questions, and 4 questions on demographics and vaccination status of the youngest child under 2 years of age in the household.

All responses were according to self-report, although DPT vaccination status was also recorded by card verification when available. Region was classified as either Region V, Region XII, or missing. The age of the respondent at the last birthday was recorded in years. The sex of the respondent was classified as male, female, or missing.

To assess proxies for socioeconomic status, we assessed educational attainment and main water source. Education was classified as did not graduate from elementary, elementary graduate, high school graduate, college graduate, postgraduate studies, or missing, and then collapsed to a binary (completed vs did not complete high school) for the analysis. Water source was classified as piped water, dug well, tube well or borehole, water from a spring, water refilling station, others, or missing.

We classified children under 2 years of age who received zero doses of DPT-containing vaccine as zero-dose, consistent with the literature [23]. Vaccination status was classified as received at least 1 dose of DPT or did not receive DPT. This variable was assessed by vaccine card verification when available in the household survey, and by self-report in all other cases (mobile and internet surveys, plus household survey respondents without available vaccine cards). Self-reported DPT vaccination status was used in the analysis, and a sensitivity analysis compared differences in DPT vaccination status between respondents who provided vaccination card verification and those who provided only self-report.

The BeSD questions were drawn from 7 of the WHO BeSD validated core questions [12] (Table S1 in Multimedia Appendix 1). We assessed confidence in vaccine importance with the question, “How important do you think vaccines are for your child’s health?” with response options of Very important, Moderately important, A little important, and Not at all important*.* Similarly, confidence in vaccine safety was assessed with the question, “How safe do you think vaccines are for your child?” with response options of Very safe, Moderately safe, A little safe, and Not at all safe. We assessed social norms with the question, “If it was time for your child to get vaccinated, would the mother need permission from any household member to take your child to the clinic?” with response options of Yes or No. In the domain of practical issues, 3 questions assessed access issues. Knowledge of where to access the vaccine was assessed with the question “Do you know where to go to get your child vaccinated?” with response options of Yes and No. Difficulty of access was assessed with the question, “How easy is it to get vaccination services for your child?” with ordinal response options of Not at all easy, A little easy, Moderately easy, and Very easy. Vaccine costs, including opportunity costs, were assessed with the question, “How easy is it to pay for vaccination?” with ordinal response options of Not at all easy, A little easy, Moderately easy, and Very easy. Vaccination intent was assessed with the question, “The Philippines has a schedule of vaccines for children. Do you want your child to get none of these vaccines, some of these vaccines or all of these vaccines?” with response options All, Some, or None.

### Statistical Analysis

Statistical analysis was conducted in Stata 18 [24].

Household survey data were analyzed using base weights for the probability of selecting each barangay and household based on the probability proportional to size, drawn from the population projections in the 2020 census. No base weights were incorporated in the mobile or online data, given the sampling design of these methods assumes uniformity across the areas. For mobile and online data, CIs are not reported, as they would not accurately reflect the true uncertainty due to the nonprobabilistic sampling approach; instead, we focus on point estimates while acknowledging the limitations in generalizability [25].

For the household, mobile, and online data, poststratification weights were used to match the marginal totals for strata defined by the combination of educational attainment of the household head, main water source, and region.

We reported respondent demographics, using the raw counts and weighted proportions for each demographic variable.

To assess the proportion of survey respondents whose children had received at least 1 dose of DPT by survey method, we calculated the poststratification weighted proportion of DPT vaccination status for each survey method, as well as the 95% CI for the household survey.

We assessed the distribution for 6 of the core BeSD questions listed above by estimating the weighted proportion of responses for each survey method. We did not assess the seventh BeSD question on vaccination intent, as intent was not directly linked with DPT vaccination status. We further explored the distribution of BeSD responses by DPT vaccination status by estimating the weighted proportion of BeSD responses by vaccination status for each survey method. We report the 95% CIs for the household survey data. We assumed missingness completely at random and used a complete records analysis.

### Process Description and Survey Costing

We describe the process of implementing each of the 3 survey methods, assessed programmatic costs, and conducted a data quality assessment for the mobile survey responses. We assessed the financial cost (monetary outlays) from the implementer’s perspective using a retrospective top-down costing approach. Measures were total cost per survey method and the cost per survey response. Financial expenses incurred by the implementer for each of the 3 survey methods were obtained from the implementer’s budget. Costs included in the analysis were all financial costs for personnel time (paid labor), per diem, transportation, equipment, BHW worker incentives, survey participant incentives, venue rental, supplies, and advertising and platform costs for each data collection method. We included costs from these program activities: planning and data collection (survey administration), cleaning, validation, and storage. Costs were assessed in 2025 Philippine Pesos (Php). We used the conversion rate on Wise on April 4, 2025 (US $1=Php 57.01) to calculate the exchange rate from Peso to US dollars, with no inflationary adjustments. These overall costs were divided by the number of survey responses completed to yield the financial cost per completed survey for each survey method. Values are presented in nominal US dollars.

The data quality assessment focused on accuracy of responses at 2 time points. First, a preprocessing assessment identified ineligible or duplicate responses automatically immediately following survey responses. Responses were deemed ineligible if the response included a respondent age below 18 years, a respondent region of residence outside of Philippines Regions V (Bicol) and XII (SOCCSKSARGEN), or a respondent-reported child age above 2 years. Second, we randomly selected 10% of mobile survey respondents for a callback to confirm eligibility. Among the mobile survey respondents who received a callback, we assessed the number flagged for quality concerns, tabulated the weighted proportion for each of the demographics and BeSD responses, and then calculated the Pearson chi-squared design-based *F* statistic and *P* values (Table S2 in Multimedia Appendix 1). These statistical analyses were possible within a single survey method, although it was not possible to conduct these analyses between the 3 survey methods, given the variations in sampling and weighting approaches. It was not possible to conduct a quality assessment for the online survey, given that we could not assess duplicate entries from the same source and contact information was not collected for follow-up verification.

### Ethical Considerations

This study was assessed and approved by the Philippines Department of Health Single Joint Ethics Review Board (SJREB-2024‐55) on November 18, 2024. This activity was reviewed by the Centers for Disease Control and Prevention (CDC)’s Human Subjects Office, deemed not research, and was conducted consistent with applicable federal law and CDC policy (see, eg, §45 CFR part 46, 21 CFR part 56; 42 USC §241(d); 5 USC §552a; 44 USC §3501 et seq). For the household survey, all participants received Php 100 (~US $1.80) to thank them for their time. For the mobile survey, participants received Php 50 (~US $0.90) upon survey completion. Participants did not receive any remuneration for their participation in the online survey.

## Results

### Respondent Characteristics

Household, mobile, and online survey methods were implemented during January to March 2025. Among the 1605 households visited for the household survey, 1208 respondents were available, of whom 1206 consented, and 1201 respondents completed the household survey (Figure S1 in Multimedia Appendix 2). Among the estimated 4451 eligible households in the participating CHW’s catchment areas, 2987 consented to participate, of whom 2500 completed the mobile survey. Following quality checks of the mobile survey data, 272 responses were excluded, including 252 perfect duplicates within the same municipality, 18 ineligible upon backchecking, and 2 with incomplete information. Among the 2135 mobile responses retained in the analytic dataset, more than 99% (2135/2153) had been completed via SMS text messaging, compared to IVR (18/2153). Among the online respondents, 26,464 initiated the survey, 996 completed it, but only 396 of them reported that they resided in the 2 eligible regions. The final analytic dataset included 1201 household survey responses, 2153 mobile responses, and 398 online responses, for a total of 3752 responses (Table 2). Excluded respondent demographics differed significantly from the analytic dataset (Table S2 in Multimedia Appendix 1).

**Table 2.** Demographics of respondents by survey method, by number and weighted proportion: Philippines Regions V (Bicol) and XII (SOCCSKSARGEN), January–March 2025.

Variables	Household		Mobile		Online	
	Value, n	Weighted % (95% CI)^a^	Value, n	Weighted %^b^	Value, n	Weighted %^b^
Region						
Region V (Bicol)	697	53.2	749	53.2	296	58.4
Region XII (SOCCSKSARGEN)	504	46.8	1404	46.8	102	41.6
Sex						
Male	55	4.5 (3.3‐6.1)	433	17.7	20	15.1
Female	1146	95.5 (93.9‐96.7)	1712	82.3	378	84.9
Missing	—^c^	—	8	—	—	—
Age (y)						
18‐25	326	29.3 (26.4‐32.3)	1078	45.4	35	13.7
26‐40	724	58.3 (54.9‐61.6)	940	48.7	342	78.6
41‐60	130	10.6 (9‐12.5)	110	5.6	18	3
>60	21	1.8 (1.2‐2.9)	7	0.3	3	4.7
Missing	—	—	18	—	—	—
Education						
Did not complete high school	321	26.8	436	37.1	17	24.5
High school graduate	877	73.2	1687	62.9	381	75.5
Missing	3	—	30	—	—	—
Main drinking water source						
Piped water	455	39	753	39	56	39.8
Tube well or borehole	133	20.1	326	20.1	11	18.2
Dug well	49	7.6	167	7.6	5	3.9
Water from a spring	83	8.9	184	8.9	26	8.7
Water refilling station	458	21.8	672	21.8	276	26.2
Others	23	2.6	51	2.6	24	3.2

a Household survey data were analyzed using base weights for the probability of selecting each barangay and household based on the probability proportional to size, drawn from the population projections in the 2020 census, along with poststratification weights to match the marginal totals of each stratum of age and sex to the regional distribution of educational attainment and main water source for each region.

b Poststratification weights were used to match the marginal totals of each stratum of age and sex to the regional distribution of educational attainment and main water source for each region. We do not report CIs for the mobile and online surveys, given that CI estimates would substantially underestimate the true variability due to their sampling approaches.

c Entries with no inputs (zero responses) and entries where weighted proportions cannot be calculated due to zero responses are indicated as “—”.

The unweighted count and weighted proportion of respondents by demographic characteristic varied substantially by survey method. The household survey had the smallest proportion of male respondents at 4.5% (n=55; 95% CI 3.3%‐6.1%), followed by the online survey at 15.1% (n=20), and the mobile survey at 17.7% (n= 433). Age also varied by survey method, with 78.6% (n=342) of online respondents reporting an age between 26 and 40 years, compared with 58.3% (n=724; 95% CI 54.9%‐61.6%) in the household survey and 48.7% (n=940) of respondents in the mobile survey. Online survey respondents were the most likely to be high school graduates at 75.5% (n=381), followed by household respondents at 73.2% (n=877), and mobile respondents at 62.9% (n=1687).

**Figure 1.**
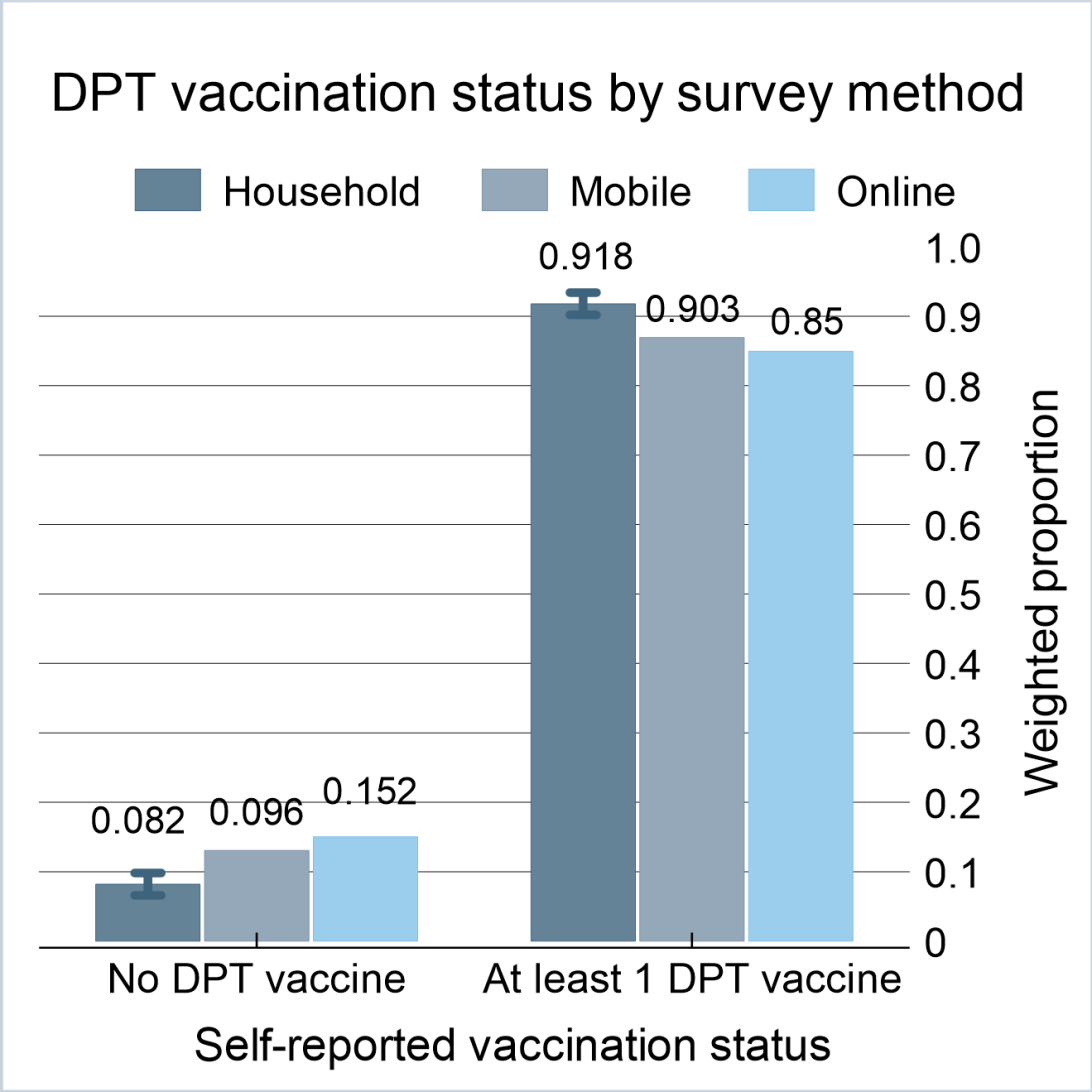
Diphtheria-pertussis-tetanus vaccination (at least 1 dose) by survey method: Philippines Regions V (Bicol) and XII (SOCCSKSARGEN), January–March 2025. DPT: diphtheria-pertussis-tetanus.

The probability of reaching a household with a child vaccinated against DPT was consistent between household and mobile surveys at 91.8% (n=1090; 95% CI 90%‐93.3%) and 90.3%, respectively, but substantially lower at 85% among online respondents (Figure 1). Among the 93.1% of household survey respondents who gave permission for vaccination card validation, 92.6% (n=1017; 95% CI 91%‐94%) of children received at least 1 dose of DPT vaccine. We found caregiver-reported DPT vaccination status (n=73, 85.2%; 95% CI 76.7%‐91%) was somewhat lower than that of those with card-verified data.

**Figure 2.**
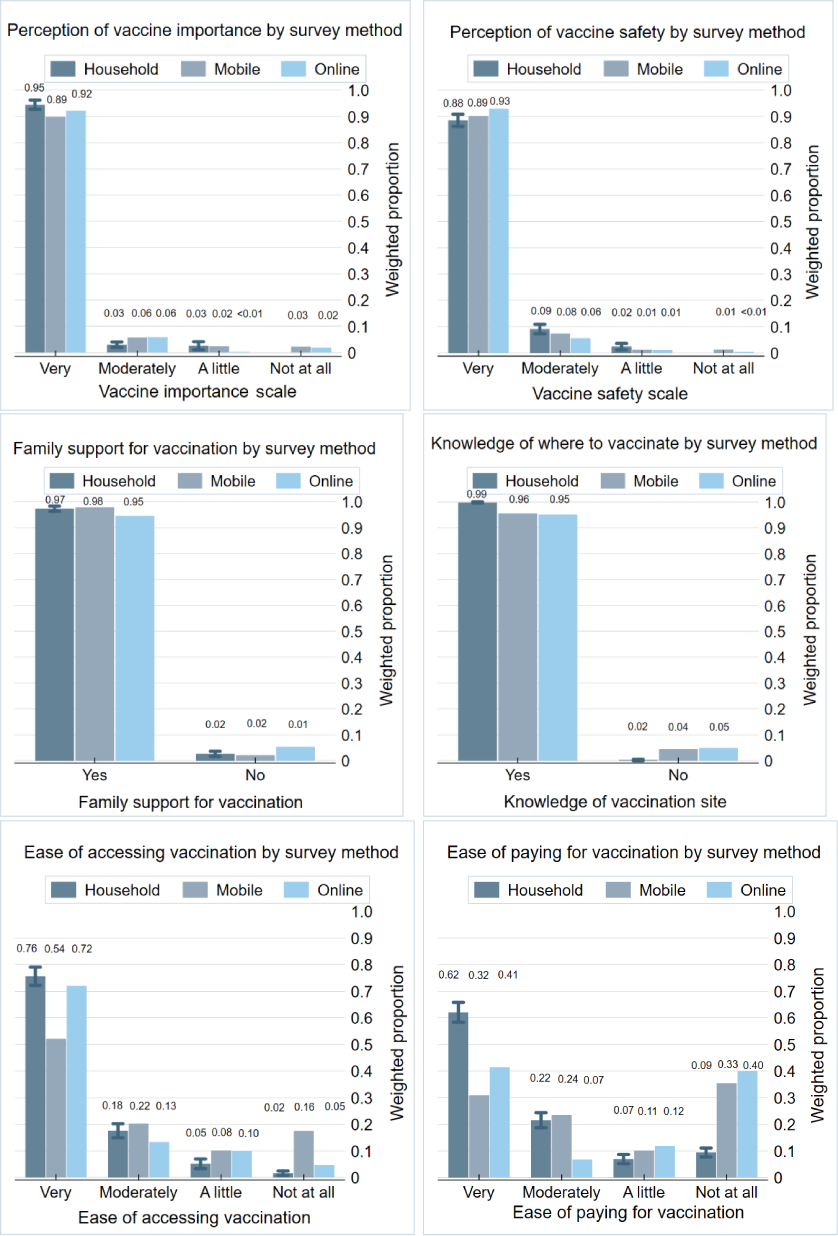
Behavioral and social drivers of vaccination by survey method: Philippines Regions V (Bicol) and XII (SOCCSKSARGEN), January–March 2025.

At least 85% of respondents in each survey method reported the highest level of vaccine demand, indicating that vaccines are very important, very safe, supported by family, and that they knew where to access vaccination (Figure 2). In response to the BeSD question on vaccine importance, 94.5% (n=1139; 95% CI 92.4%‐96%) of household survey respondents, 89.3% (n=2258) of mobile respondents, and 92.2% (n=381) of online respondents indicated that vaccines are very important. In response to the BeSD question on vaccine safety, 88.5% (n=1056; 95% CI 86%‐90.6%) of household survey respondents, 89.7% (n=2240) of mobile respondents, and 92.8% (n=386) of online respondents indicated that vaccines are very safe. In response to the BeSD question on family support for vaccination, 97.3% (n=1166; 95% CI 96.1%‐98.2%) of household survey respondents, 97.8% (n=2449) of mobile respondents, and 94.6% (n=390) of online respondents indicated that their families supported vaccination. In response to the BeSD question on knowing where to access vaccination, 99.8% (n=1198; 95% CI 99.2%‐99.9%) of household survey respondents, 96.3% (n=2362) of mobile respondents, and 95.1% (n=393) of online respondents indicated that they knew where to access vaccination.

Reported ease of accessing vaccination varied by survey method, with the highest proportion of mobile survey respondents indicating that it was not at all easy to access vaccination (Figure 2). Among household survey respondents, 75.6% (n=899; 95% CI 72.1%‐78.9%) indicated it was very easy to access vaccination, compared with 53.9% (n=1205) of mobile respondents and 72% (n=294) of online respondents. Similarly, reported ease of paying for vaccination varied by survey method, with more than 30% of mobile and online survey respondents indicating it was not at all easy to pay for vaccination, compared with 9.4% (n=112; 95% CI 7.9%‐11.2%) of household respondents. Conversely, 62.1% (n=754; 95% CI 58.3%‐65.7%) of household survey respondents indicated that it was very easy to pay for vaccination, compared with 32.1% (n=744) of mobile respondents and 41.4% (n=169) of online respondents.

The responses to BeSD questions on vaccine importance, vaccine safety, and family support for vaccination did not vary substantially by survey method when the distribution of responses was categorized by DPT vaccination status (Figure S2 in Multimedia Appendix 3).

### Process Description

Among the 2153 responses retained in the analytic dataset for the mobile survey, 348 mobile survey respondents received a quality callback, of whom 117 (33.6%)o JMIR reviewer callbacks indicated that the initial survey response was ineligible or not concordant with the original responses to demographic survey questions. The demographic distribution of the respondents flagged for quality concerns differed significantly from the demographic distributions of those not flagged for quality concerns (Table S2 in Multimedia Appendix 1). However, the BeSD responses for vaccine importance, safety, family support for vaccination, and ease of paying for vaccination did not vary significantly between responses flagged for quality concerns and those that were not flagged for quality concerns (Table S2 in Multimedia Appendix 1).

The financial cost per survey from the implementer’s perspective, in nominal US $, was US $2.10 per survey response for the online survey, US $6.93 for the mobile survey, and US $29.38 for the household survey (Table S3 in Multimedia Appendix 1). Personnel time paid labor and per diem were the main cost drivers for the household survey, respondent incentives were the main cost driver for the mobile survey, and advertising and platform costs were the main cost drivers for the online survey.

## Discussion

### Principal Findings

Our study comparing 3 different methods of collecting BeSD of vaccination data in 2 regions of the Philippines found that respondent characteristics varied by method, with mobile survey respondent demographics and child vaccination status closer to household survey responses than online survey responses. Of particular interest, the proportion of caregivers reporting a child unvaccinated against DPT was 9.7% for the household survey, 8.2% for the mobile survey, and 15% for the online survey, indicating that all 3 survey methods were able to access unvaccinated populations, with online respondents reporting the highest levels of unvaccinated children.

Overall, respondents for each survey method indicated high vaccine demand, with more than 85% of respondents in each survey method indicating that vaccines are very important, very safe, supported by family, and that the site to access vaccination was known. Within these overall trends, the mobile survey responses were more closely reflective of the household responses than the online survey responses. However, responses to questions on ease of accessing and paying for vaccination illustrated more heterogeneity in survey responses and between survey methods, with household survey respondents more likely to indicate that it was very easy to access and pay for a vaccine than mobile and online survey respondents. These differential responses to the BeSD questions on challenges could be due to underlying differences in population characteristics, or they could be attributed to differential effects of social desirability bias between the in-person household survey and the self-administered mobile and online surveys.

The cost per survey response increased threefold from online to mobile and fourfold from mobile to household survey. A quality check reassessed 10% of mobile survey responses through callbacks and identified 37 instances of inconsistent responses; a quality assessment did not identify substantive differences in magnitude or significance for 4 of the core BeSD questions between responses flagged in sensitivity checks and those that were not flagged.

Each survey method offered benefits and drawbacks. The household survey generated responses that could be considered representative of their respective regions, though the costs per response were considerably higher than the other 2 methods. The mobile survey could be conducted at a fraction of the cost of the household survey, and it yielded similar responses to the BeSD questions. However, the demographic distribution of the respondents differed substantially from that of the household survey, and the quality assessment identified nearly one-third of the responses as ineligible, based on region or caregiver status. The online survey was the least expensive to implement and also yielded similar responses to the BeSD questions as the other 2 methods. However, online survey respondent demographics were substantially different from those of household survey respondents, and there was no means to ascertain respondent eligibility by regional residence or caregiver status.

The WHO has recommended that all countries incorporate questions from BeSD validated surveys [26] in routine data collection and recommends that those with low or inequitable coverage of childhood immunization conduct national or subnational surveys every 2 to 3 years [27]. These indicators have been deployed in contexts ranging from human papillomavirus vaccination in Zimbabwe [28] to COVID-19 vaccination in the Philippines [29]. However, the routine implementation of these validated survey questions poses challenges in resource-constrained settings, particularly if rapid data collection is needed following an event that causes a rapid drop in vaccine confidence.

Our work adds to the growing literature that compares mobile and online data collection methods to household surveys in LMICs [30]. A Cochrane review identified app-based data collection methods as noninferior to other methods of data collection [31], but there were no studies included from LMICs. In a systematic review comparing survey methods in LMICs, only 3 of the included studies compared remotely delivered surveys to household surveys, of which 2 focused on infant feeding programs [31,32] and a third examined household poverty levels following economic shocks. To our knowledge, this study is the first comparison of survey methods regarding BeSD of vaccination at the community level in an LMIC.

Compared to mobile and online methods, household surveys are typically the most representative of population demographics, with mobile and online data drawing a relatively lower proportion of older respondents, rural respondents, and those with less than a high school education and less relative wealth [10,33,34]. Our study identified similar trends in representativeness by survey method [35], although the key variable of interest, DPT vaccination status, differed between data collection methods, indicating that mobile and online surveys are less reliable than household surveys in ascertaining vaccination status.

The literature suggests that the reliability of responses by survey method depends on the type of question asked [36], though mobile surveys have been demonstrated to be noninferior to in-person data collection in survey responsiveness for questions deemed less sensitive [37]. An analysis comparing sequential calling of IVR and computer-assisted telephone interviewing in Bangladesh and Tanzania found high consistency in response to questions on alcohol consumption type and smoking, moderate consistency for history of diabetes or hypertension, and low consistency for routine activities, including physical activity and diet [38]. In a study comparing farmers in India who were surveyed both in person and by phone, significant differences in production were detected due to differential answers by survey method, rather than differential attrition [33]. Our study identifies inconsistencies in demographics by survey method but identifies similarities in perceptions of vaccine importance, safety, family support, and knowledge of where to access vaccination across survey methods.

We found that the financial cost per survey method differed substantially, with the online survey the least expensive and the household survey the most. These findings are consistent with other studies that evaluate costs per survey method between SMS text messaging, IVR, and computer-assisted telephone interviewing [39] and those that examine costs of including mobile phones in existing survey modalities [40]. Our direct comparison by survey method allows decision-makers to assess the preferred survey method in the context of representativeness, responses to core BeSD questions, and financial costs from the implementer’s perspective.

To our knowledge, our study represents the first direct comparison of different survey methods for BeSD data. The implications of our study illustrate that while the survey respondents substantially differ by data collection method, responses to the BeSD questions were markedly similar across all 3 methods. These findings point to the potential of mobile and online methods to complement household surveys, particularly in contexts where mobile ownership or internet connectivity is high and there is reported homogeneity in vaccine drivers across subpopulations.

### Limitations

This study has at least 5 limitations. First, the survey methods did not use the same sampling frame; thus, it was not possible to assess how the underlying population demographics varied between the 3 sampling frames. The differences in sampling frames limit our ability to assess the extent to which differences between surveys were attributable to the underlying population differences in sampling frames vs the differences in respondents by survey type. We aimed to address this limitation by sampling adjacent barangays for the household and mobile survey methods, and we adjusted for key demographic and socioeconomic factors. In addition, we do not draw conclusions on comparative representativeness by survey type due to this limitation. Second, our data do not permit an assessment of response rates for each data method, as the denominator for the mobile surveys and online data was not available. We report only the number of responses and compare the representativeness of respondents between the survey methods. Third, with the exception of vaccination card verification in the household survey method, the responses to the survey questions could not be independently verified and could have been subject to social desirability bias or recall limitations. To help mitigate this, in-person enumerators assured caregivers that responses were anonymous and, when possible, referred to the child’s vaccination card for verification. While the BeSD questions cannot be independently verified, we sought to mitigate any unmeasured social desirability bias through the remote administration methods and by training household survey data collectors in appropriate data collection techniques that mitigate the risk of these biases. In addition, the mobile survey method included a data quality assessment of response accuracy to quantify the proportion of respondents whose responses to demographic questions indicated that they were not eligible by age, region of residence, or being caregivers of children 2 years of age or younger. Fourth, by distributing the mobile survey by BHWs, the mobile survey sampling frame was not structured to reach households that might be missed by household survey methods and could therefore be subject to similar biases in missing transitory or hard-to-reach populations. Furthermore, the mobile survey distribution via BHWs also limits the generalizability of these findings to contexts where trusted community health workers maintain listings of vaccine-eligible households in their catchment areas. While we acknowledge this limitation, we also note that the mobile methods yielded a similar proportion of children unvaccinated against DPT. Finally, we note that the financial costing of survey administration from the implementer’s perspective does not include the economic costs, such as the in-kind labor cost of workers whose salaries are covered by the Department of Health, or the systems cost of integrating BeSD data collection into Department of Health data platforms. Furthermore, financial costs presented here are those reported by the implementer on its budgets and may differ from the actual expenses incurred. Similarly to the survey response rates, costs of each survey method are not directly comparable because their representativeness and length differ.

### Conclusions

Although respondent demographics and child vaccination status differed between household, mobile, and online data collection methods, respondents consistently indicated high vaccine demand as well as challenges in accessing vaccination and in the cost of accessing vaccination. Selecting the most suitable survey method requires a consideration of trade-offs between methods, including cost, access to technology, and target demographics of caregivers or children. In areas with high mobile phone ownership and strong mobile and internet connectivity, routine mobile and online vaccine demand data collection methods can supplement periodic household surveys.

## Supplementary material

10.2196/81059 Multimedia Appendix 1 Survey questions and response options for each survey method, mobile survey quality assessment, and total financial cost and financial cost per survey response.

10.2196/81059 Multimedia Appendix 2 Flow diagram of household, mobile, and online survey participants: Philippines Regions V (Bicol) and XII (SOCCSKSARGEN), January–March 2025.

10.2196/81059 Multimedia Appendix 3 Behavioral and social drivers of vaccination by DPT vaccination status and survey method: Philippines Regions V (Bicol) and XII (SOCCSKSARGEN), January–March 2025.

## Abbreviations

BeSD: behavioral and social drivers
BHW: Barangay Health Worker
CDC: Centers for Disease Control and Prevention
DOH: Department of Health
DPT: diphtheria-pertussis-tetanus
IVR: interactive voice response
LMIC: low- and middle-income country
