# Supplementary material for: Child Vaccination Status and Behavioral and Social Drivers of Vaccination Among Their Caregivers in the Philippines: Cross-Sectional Survey Study Comparison of Household, Mobile, and Online Modes
Source: J Med Internet Res. 2026 Apr 10;28:e81059. doi: 10.2196/81059 (PMC13068193; doi:10.2196/81059)
Supplement: Multimedia Appendix 1 [file jmir-v28-e81059-s001.docx]

# Supplementary tables

#

Table S1. Survey questions and response options for each survey method: Philippines Regions V (Bicol) and XII (SOCCSKSARGEN) January—March 2025

| Question | Responses | Household | Mobile | Online |
| --- | --- | --- | --- | --- |
| I. Introduction and Informed Consent Form |  | X | X | X |
| II. Respondent Profile - Caretaker |  |  |  |  |
| What is your age as of your last birthday? | _______ Years | X | X | X |
| What is your sex? | Female  Male | X | X | X |
| What is your current marital status? | Single / Never Married  Married  Common law / Live-in  Widowed  Divorced  Separated  Annulled  Not reported  Refuse to answer [DO NOT READ OUT LOUD.] | X |  |  |
| During the past 12 months, what was your usual activity/occupation? | Has a permanent job / business  Has a short-term, seasonal, casual job / business  Worked on different jobs day to day per week  Unemployed and looking for work  Unemployed and not looking for work  Retired  I don't know  Not applicable  Refuse to answer [DO NOT READ OUT LOUD.] | X |  |  |
| Where do you get your personal income? Please don't include income generated by other people. | Working for private company  Working for private household  Working for government  Worked with pay in own family business or farm  Self-employed without any paid employee  Employer in own family business  Worked without pay in own family business or farm  Pension  Unemployed and looking for work  Unemployed and not looking for work  I don't know  Refuse to answer [DO NOT READ OUT LOUD.] | X |  |  |
| How many people are there in your household?  Please count yourself and all the people who usually live with you, including children below 18 y/o. Please include those who sleep and eat from the same pot here at least once a week, BUT do not include OFWs. | __________ | X |  |  |
| What are the construction materials of the roof of your dwelling? | No roof  Thatch/Palm leaf  Sod  Rustic mat  Palm/bamboo  Wood planks  Cardboard  Metal  Calamine / Cement Fiber  Ceramic Tiles  Cement  Roofing Shingles  Other (specify):  Refuse to answer [DO NOT READ OUT LOUD] | X |  |  |
| What are the construction materials of the outer walls of your dwelling? | No walls  Cane/Palm/Trunks  Dirt  Bamboo with Mud  Stone with Mud  Uncovered adobe  Plywood  Cardboard  Reused wood  Cement  Stone with Lime/Cement  Bricks  Covered adobe  Wood planks/ Shingles  Other (specify):  Refuse to answer [DO NOT READ OUT LOUD] | X |  |  |
| What is the main source of drinking water for members of your household? | Piped into dwelling  Piped to yard/plot  Piped to neighbor  Public tap/standpipe  Tube well or borehole  Protected well  Unprotected well  Protected spring  Unprotected spring  Rainwater  Tanker truck  Cart with small tank  Surface Water (River/Dam/Lake/Pond/Stream)  Bottled water  Refuse to answer [DO NOT READ OUT LOUD.] | X |  |  |
| Do you have your own faucet or do you share with your community? | I/we have our own  I/we share with our community  Refuse to answer [DO NOT READ OUT LOUD] | X |  |  |
| Do you have your own tube/pipe or do you share with your community? | I/we have our own  I/we share with our community  Refuse to answer [DO NOT READ OUT LOUD] | X |  |  |
| Does your household have electricity? | Yes  No  Refuse to answer [DO NOT READ OUT LOUD.] | X |  |  |
| Does your household have a refrigerator / freezer? | Yes  No  Refuse to answer [DO NOT READ OUT LOUD.] | X |  |  |
| Does your household have a television set? | Yes  No  Refuse to answer [DO NOT READ OUT LOUD.] | X |  |  |
| Does your household have a washing machine? | Yes  No  Refuse to answer [DO NOT READ OUT LOUD.] | X |  |  |
| What is the highest grade/year completed by your head of the household? | Early Childhood Education (Pre-school)  Primary Education (Grade 1 to 6)  Lower Secondary Education (Grade 7 to 10)  Upper Secondary Education (Grade 11 to 12)  Post-Secondary Non-Tertiary Education (including Technical and Vocational degrees with a certificate)  Short-Cycle Tertiary Education or Equivalent (including Technical and Vocational degrees with a diploma)  Bachelor Level Education or Equivalent  Master Level Education or Equivalent  Doctoral Level Education or Equivalent  No schooling  Other (specify)  I don't know  Not applicable  Refuse to answer [DO NOT READ OUT LOUD.] | X | X | X |
| III. Child Profile |  |  |  |  |
| How old is your child? | 0 to 6 months  7 months to 12 months  13 months to 24 months  Refuse to answer [DO NOT READ OUT LOUD.] | X | X | X |
| What is the sex of your youngest child? | Female  Male | X |  |  |
| The Philippines has a vaccination schedule for children. As far as you know, has your youngest child between the age of 0-2 received none, some, or all of these vaccines? | None  Some  All  Refuse to answer [DO NOT READ OUT LOUD.] | X | X | X |
| IV. BeSD Survey |  |  |  |  |
| The Philippines has a schedule of vaccines for children. Do you WANT your child to get none of these vaccines, some of these vaccines or all of these vaccines? | None  Some  All  Refuse to answer [DO NOT READ OUT LOUD.] | X | X | X |
| Why not? | Clinic is too far  Clinic takes too long  I want to do it when my child is older  I don't think vaccines are safe  I don't think vaccines are important  My family (including husband/partner) does not want  My child was unwell on the appointment data  I am still not sure if I want my child vaccinated  Other (specify)  Refuse to answer [DO NOT READ OUT LOUD.] | X |  |  |
| Other (specify) | _____________ | X |  |  |
| How effective do you think vaccines are in reducing your child's likelihood of getting infectious diseases, such as TB, pneumonia, among many others. | Not at all effective  A little effective  Moderately effective  Very effective  Refuse to answer [DO NOT READ OUT LOUD.] | X |  |  |
| How important do you think vaccines are for your child’s health? Would you say... | Not at all important  A little important  Moderately important  Very important  Refuse to answer [DO NOT READ OUT LOUD.] | X | X | X |
| How safe do you think vaccines are for your child? Would you say... | Not at all safe  A little safe  Moderately safe  Very safe  Refuse to answer [DO NOT READ OUT LOUD.] | X | X | X |
| How much do you trust the health workers who give children vaccines? Would you say you trust them... | Not at all  A little  Moderately Very  Refuse to answer [DO NOT READ OUT LOUD.] | X |  |  |
| Do you think most parents you know get their children vaccinated? | Yes  No  Refuse to answer [DO NOT READ OUT LOUD.] | X |  |  |
| Do you think most of your close family and friends want you to get your child vaccinated? | Yes  No  Refuse to answer [DO NOT READ OUT LOUD.] | X | X | X |
| Do you think your religious leaders want you to get your child vaccinated? | Yes  No  Refuse to answer [DO NOT READ OUT LOUD.] | X |  |  |
| Do you think your community leaders want you to get your child vaccinated? | Yes  No  Refuse to answer [DO NOT READ OUT LOUD.] | X |  |  |
| Has a health worker recommended your child be vaccinated? | Yes  No  Refuse to answer [DO NOT READ OUT LOUD.] | X |  |  |
| Have you ever been contacted about your child being due for vaccination? | Yes  No  Refuse to answer [DO NOT READ OUT LOUD.] | X |  |  |
| If it was time for your child to get vaccinated, would the mother need permission from any household member to take your child to the clinic? | Yes  No  Refuse to answer [DO NOT READ OUT LOUD.] | X |  |  |
| Do you know where to go to get your child vaccinated? | Yes  No  Refuse to answer [DO NOT READ OUT LOUD.] | X | X | X |
| Have you personally ever taken your child to get vaccinated? | Yes  No  Refuse to answer [DO NOT READ OUT LOUD.] | X |  |  |
| Have you ever been turned away when you tried to get your child vaccinated? | Yes  No  Refuse to answer [DO NOT READ OUT LOUD.] | X |  |  |
| What was the reason for being turned away? | Not enough vaccine supply  Not the right vaccine schedule  No healthcare worker/midwife to administer the vaccine  No reason given  Other (specify)  Refuse to answer [DO NOT READ OU T LOUD.] | X |  |  |
| How easy is it to get vaccination services for your child? Would you say... | Not at all easy  A little easy  Moderately easy  Very easy  Refuse to answer [DO NOT READ OUT LOUD.] | X | X | X |
| What makes it hard to get vaccination services for your child? Would you say... | Nothing, it’s not hard  Getting to the clinic is hard  The clinic opening times are inconvenient  The clinic sometimes turns people away without vaccinating  The waiting time in the clinic takes too long  Other (please specify):  Refuse to answer [DO NOT READ OUT LOUD] | X |  |  |
| How easy is it to pay for vaccination? When you think about the cost, please consider any payments to the clinic, the cost of getting there, plus the cost of taking time away from work. Would you say... | Not at all easy  A little easy  Moderately easy  Very easy  Refuse to answer [DO NOT READ OUT LOUD.] | X | X | X |
| Which of the following costs made it most difficult to pay for vaccination? | Payment to health facility for services  Payment for vaccines  Cost to travel to health facility / vaccination site  Cost to take time off work  Refuse to answer [DO NOT READ OUT LOUD.] | X |  |  |
| How satisfied are you with the vaccination services? Would you say... | Not at all satisfied  A little satisfied  Moderately satisfied  Very satisfied  Not applicable, I have never tried to get my child vaccinated  Refuse to answer [DO NOT READ OUT LOUD.] | X |  |  |
| What is not satisfactory about the vaccination services? Would you say... | Nothing, you are satisfied  Vaccine is not always available  The clinic does not open on time  Waiting times are long  The clinic is not clean  Staff are poorly trained  Staff are not respectful  Staff do not spend enough time with people  Other (please specify):  Not applicable, I have never tried to get my child vaccinated  Refuse to answer [DO NOT READ OUT LOUD.] | X |  |  |
| Other (specify) | ______________ |  |  |  |
| V. Vaccination Status |  |  |  |  |
| Do you have a card where your child's vaccinations are written down? | Yes  No | X |  |  |
| May I see the vaccination card of your child? | Yes  No | X |  |  |
| Which of the following vaccinations has the youngest child aged 0-2 received? |  | X |  |  |
| Please input how many times a OPV was received: | ____________ | X |  |  |
| Please input how many times IPV was received: | ____________ | X |  |  |
| Please input how many times a DPT vaccination was received:    In the SMS/IVR and online surveys: Has your child received the pentavalent vaccine, a shot given in the thigh, more than a month after birth? | ____________ | X | X | X |
| Please input how many times a Hepatitis B vaccination was received:  EXCLUDE THE DOSE RECEIVED AT BIRTH | ____________ | X |  |  |
| Please input how many times a PCV vaccination was received: | ____________ | X |  |  |
| Please input how many times a MMR vaccination was received: | ____________ | X |  |  |

Table S2. Mobile survey quality assessment: comparison of demographic and BeSD responses by survey flag, Philippines Regions V (Bicol) and XII (SOCCSKSARGEN), January—March 2025

|  | Pre-processing assessment | | Pearson Design-based F-statistic and P value | 10% callback post-assessment | | Pearson Design-based F-statistic and P value |
| --- | --- | --- | --- | --- | --- | --- |
|  | Not flagged | Flagged |  | Not flagged | Flagged |  |
| Number | 2,153 | 347 |  | 231 | 117 |  |
| Region |  |  |  |  |  |  |
| Region V: | 96.0 | 4.1 | F(1, 2467)= 39.50; P= 0.000 | 80.0 | 20.0 | F(1, 344) = 22.57; P = 0.000 |
| Region XII: | 81.9 | 18.1 |  | 56.1 | 43.9 |  |
| Sex |  |  |  |  |  |  |
| Male | 84.2 | 15.8 | F(1, 2461) = 12.82; P=0.0003 | 39.6 | 60.4 | F(1, 343) = 25.297; P = 0.000 |
| Female | 90.4 | 9.6 |  | 74.1 | 25.9 |  |
| Age |  |  |  |  |  |  |
| 18-25 | 83.0 | 17.0 | F(1.92, 4698.56)= 16.72; P= 0.000 | 61.0 | 39.0 | F(1.90, 648.68) = 7.99; P = 0.0005 |
| 26-40 | 94.1 | 6.0 |  | 72.6 | 27.4 |  |
| 41-60 | 99.0 | 1.0 |  | 93.4 | 6.6 |  |
| >60 | 100 | 0 |  | 68.8 | 3.1 |  |
| Educational attainment | | | | | | |
| Did not complete HS | 87.4 | 12.6 | F(1, 2467)= 3.52; P=0.0606 | 61.8 | 38.2 | F(1, 344)= 4.89; P = 0.028 |
| HS Graduate | 90.5 | 9.5 |  | 72.8 | 27.2 |  |
| Main water source | | | | | | |
| Piped water | 85.0 | 15.0 | F(2.47, 6082.77)= 7.33; P=0.0002 | 68.7 | 31.3 | F(3.42, 1177.73)= 4.39; P=0.0028 |
| Tube well | 92.4 | 7.7 |  | 65.7 | 34.3 |  |
| Dug well | 85.1 | 14.9 |  | 42.6 | 57.4 |  |
| Spring Water | 86.8 | 13.2 |  | 66.4 | 33.7 |  |
| Refilling station | 97.0 | 3.0 |  | 81.1 | 18.9 |  |
| Others | 89.8 | 10.2 |  | 79.9 | 20.2 |  |
| DPT vaccination status | | | | | | |
| No DPT | 60.2 | 39.8 | F(1, 2398)= 231.28; P =0.00 | 33.1 | 66.9 | F(1, 328) = 19.17; P =0.000 |
| DPT | 93.5 | 6.5 |  | 71.8 | 28.2 |  |
| Vaccine importance |  |  |  |  |  |  |
| Very | 88.8 | 11.2 | F(2.31, 5707)= 2.04; P=0.123 | 68.8 | 31.2 | F(2.77, 954.55) =0.352; P=0.77 |
| Moderate | 92.7 | 7.4 |  | 64.5 | 35.5 |  |
| A little | 95.5 | 4.5 |  | 68.3 | 31.7 |  |
| Not at all | 97.6 | 2.5 |  | 83.0 | 17.0 |  |
| Vaccine safety |  |  |  |  |  |  |
| Very | 89.0 | 11.0 | F(2.54, 6275.10)=0.84; P = 0.457 | 70.2 | 29.8 | F(2.91, 1001.24)= 0.89; P = 0.443 |
| Moderate | 92.5 | 7.5 |  | 60.5 | 39.5 |  |
| A little | 94.7 | 5.3 |  | 65.3 | 34.7 |  |
| Not at all | 89.8 | 10.2 |  | 43.7 | 56.3 |  |
| Family support for vaccines | | | | | | |
| Yes | 89.3 | 10.8 | F(1, 2466) = 1.31; P =0.252 | 69.2 | 30.9 | F(1, 343) = 1.08; P = 0.299 |
| No | 94.6 | 5.3 |  | 49.0 | 51.5 |  |
| Knows where to vaccinate | | | | | | |
| Yes | 90.4 | 9.6 | F(1, 2467) = 54.42; P =0.00 | 69.9 | 30.1 | F(1, 344) = 5.093; P = 0.025 |
| No | 67.5 | 32.5 |  | 40.2 | 59.8 |  |
| Ease of accessing vaccines | | | | | | |
| Very | 94.0 | 6.0 | F(2.74, 6770.69)= 50.208; P = 0.000 | 71.5 | 28.5 | F(2.96, 1019.35)=3.73; P=0.011 |
| Moderate | 97.3 | 2.6 |  | 79.7 | 20.3 |  |
| A little | 67.4 | 32.6 |  | 51.1 | 48.9 |  |
| Not at all | 78.8 | 21.2 |  | 56.7 | 43.4 |  |
| Ease of paying for vaccines | | | | | | |
| Very | 93.8 | 6.2 | F(1.90, 4681.18)= 17.356 P = 0.000 | 73.1 | 26.9 | F(2.98, 1024.16)= 0.957; P = 0.412 |
| Moderate | 95.0 | 5.0 |  | 69.4 | 30.6 |  |
| A little | 94.8 | 5.2 |  | 59.5 | 40.5 |  |
| Not at all | 80.2 | 19.8 |  | 66.4 | 33.6 |  |

Table S3. Total financial cost and financial cost per survey response in nominal U.S. Dollars by survey method: Philippines Regions V (Bicol) and XII (SOCCSKSARGEN), January—March 2025

| **Costs** | **Definition** | **Household** | **Mobile** | **Online** |
| --- | --- | --- | --- | --- |
| **Data collection costs** |  |  |  |  |
| Personnel time paid labor* | Allocation of labor to design and implement data collection activities | $10,689.84 | $1,901.75 |  |
| Per diem | Any allowance paid to workers for data collection activities | $12,775.41 | $1,965.93 |  |
| Transportation | Cost of care hire, bus fare, plane travel | $5,625.29 | $1,304.62 |  |
| Equipment | Purchase of communication and data-related equipment and devices | $0.00 | $0.00 |  |
| BHW worker incentive | Allowance paid to BHWs for participation | $2,107.02 | $2,702.40 |  |
| Venue | Space rented for training or meetings, or office space | $0.00 | $1,578.95 |  |
| Supplies | Purchase of stationery and office supplies | $2,237.72 | $0.00 |  |
| **Advertising and platform costs** |  | $0.00 | $8,118.07 | $835.47 |
| **Total Cost** |  | $35,283.06 | $14,928.75 | $835.47 |
| **Number of completed surveys** |  | 1201 | 2153 | 398 |
| **Cost per survey** |  | $29.38 | $6.93 | $2.10 |

*Labor cost for the online survey is zero because it was designed and implemented by the Department of Health using a pre-existing online platform. Therefore, financial cost for the implementer is zero.
