# Supplementary figures and images for: Child Vaccination Status and Behavioral and Social Drivers of Vaccination Among Their Caregivers in the Philippines: Cross-Sectional Survey Study Comparison of Household, Mobile, and Online Modes
Source: J Med Internet Res. 2026 Apr 10;28:e81059. doi: 10.2196/81059 (PMC13068193; doi:10.2196/81059)

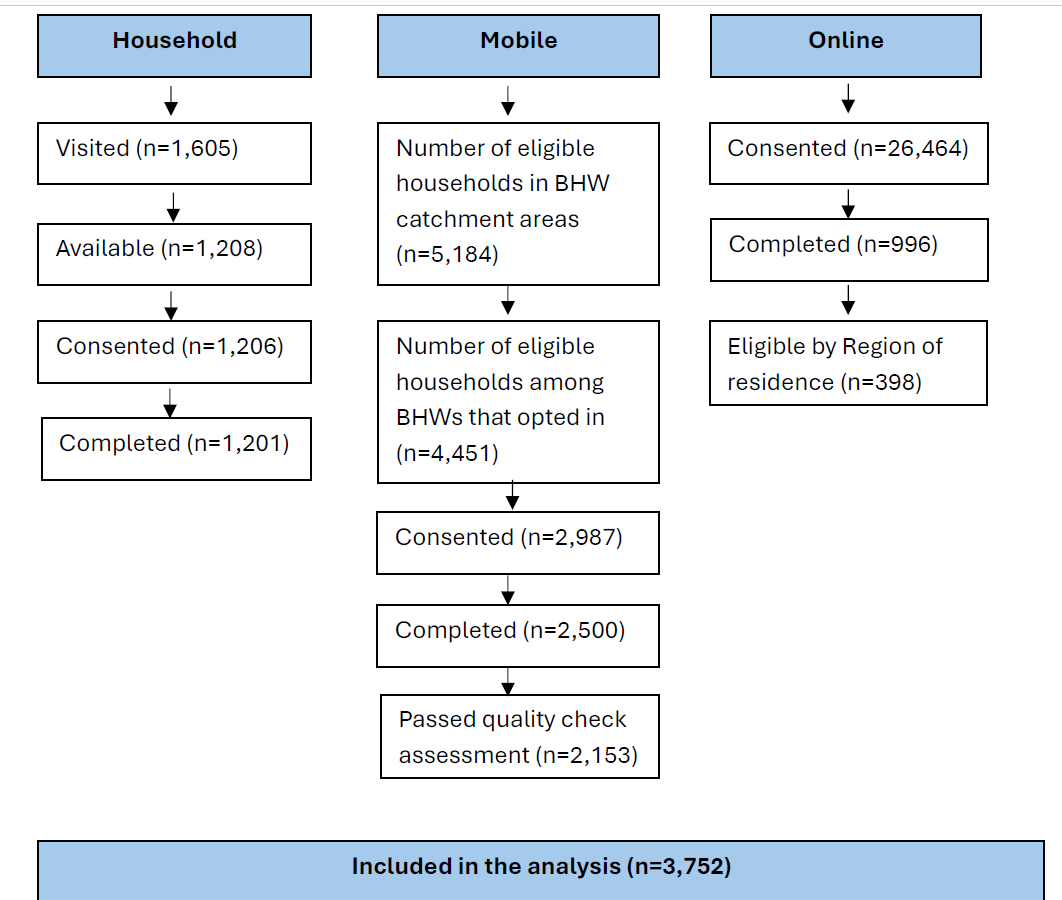

Supplement: Multimedia Appendix 2 [file jmir-v28-e81059-s002.png]

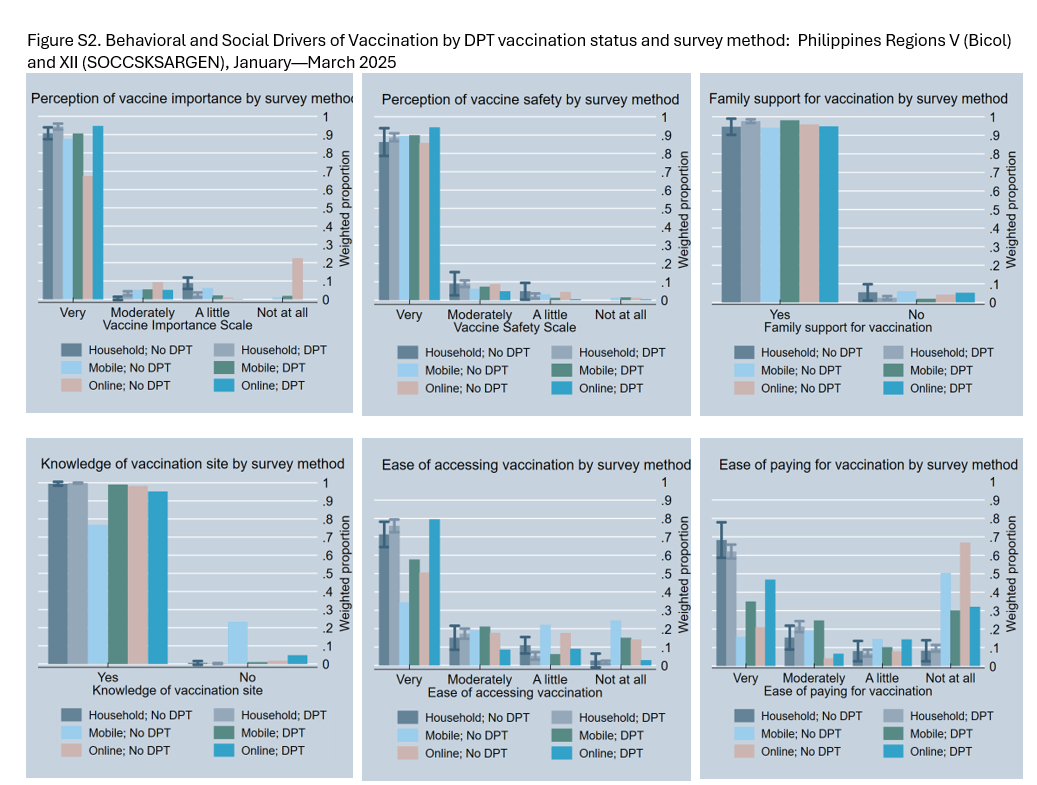

Supplement: Multimedia Appendix 3 [file jmir-v28-e81059-s003.png]
